# Knowledge and Attitudes Toward Obstructive Sleep Apnea Among Korean Pulmonologists: A Nationwide Survey

**DOI:** 10.7759/cureus.61747

**Published:** 2024-06-05

**Authors:** Kyu Yean Kim, Hyeon Hui Kang, Young-Jae Cho, Sang-Ha Kim, Sang Haak Lee, Sei Won Kim

**Affiliations:** 1 Department of Internal Medicine, Uijeongbu St. Mary’s Hospital, College of Medicine, The Catholic University of Korea, Uijeongbu, KOR; 2 Department of Internal Medicine, Eunpyeong St. Mary’s Hospital, College of Medicine, The Catholic University of Korea, Seoul, KOR; 3 Department of Internal Medicine, Seoul National University, Bundang Hospital, Seongnam, KOR; 4 Department of Internal Medicine, Wonju College of Medicine, Yonsei University, Wonju, KOR

**Keywords:** korea, attitude, knowledge, pulmonology, obstructive sleep apnea

## Abstract

Background: Obstructive sleep apnea (OSA) significantly impacts cardiovascular, metabolic, and respiratory health. In Korea, OSA patients are treated by specialists in internal medicine, otolaryngology, neurology, and psychiatry, but the participation rate of pulmonologists in OSA management is relatively low compared to other specialties. This study investigated the knowledge and attitudes about OSA among Korean pulmonologists.

Materials and methods: An online survey was conducted, targeting respiratory specialists listed in the Korean Academy of Tuberculosis and Respiratory Diseases directory. The survey used the validated "Obstructive Sleep Apnea Knowledge and Attitudes" (OSAKA) questionnaire, which consists of questions about knowledge and attitudes on OSA. To maximize participation, email invitations were sent three times to the target audience.

Results: Out of 634 queried pulmonologists, 127 (20%) responded to the survey. The mean age of respondents was 45.4 ± 8.6 years. The respondents' years of specialty acquisition ranged from the 1980s to the 2010s. Additionally, 74 (58.3%) held a doctor’s degree, and 96 (75.6%) worked in hospitals with a sleep center. Furthermore, 71 (55.9%) of the pulmonologists reported having experience with OSA patients. Pulmonologists with experience managing OSA patients had significantly higher knowledge and attitude scores compared to those without such experience. Interestingly, older respondents and those who completed their pulmonology training earlier had higher attitude scores. In addition, the knowledge score significantly correlated with responses to the five items of the attitude questionnaire.

Conclusion: This study provides valuable insights into the knowledge and attitudes of Korean pulmonologists regarding OSA. The findings indicate that their knowledge levels are comparable to or better than those in previous studies. These results underscore the need for targeted educational programs and practical training, especially for younger pulmonologists, to enhance their proficiency in managing OSA and to encourage a more active role in its treatment.

## Introduction

Obstructive sleep apnea (OSA) is a common disease that develops in 4%-10% of adults and is associated with significant morbidity and mortality [1]. Globally, the prevalence of OSA is 20%-50% among those aged ≥65 years and >40% in the obese population [2,3]. OSA is associated with hypertension, diabetes mellitus, atrial fibrillation, heart failure, coronary heart disease, stroke, and death [4]. The relationship between OSA and cardiovascular diseases has frequently been reported by epidemiological and clinical studies [4,5]. Intermittent hypoxemia, sympathetic activation, oxidative stress, and inflammation have been proposed as the underlying mechanisms of OSA onset [6-8]. The effects of OSA on pulmonary diseases, such as chronic obstructive pulmonary disease (COPD) and idiopathic pulmonary fibrosis, have also been studied [9,10].

Despite its high prevalence and clinical significance, OSA is often underdiagnosed. This is probably because many primary care physicians are not familiar with OSA. In 2003, Helena et al. developed a questionnaire, known as the Obstructive Sleep Apnea Knowledge and Attitudes (OSAKA) questionnaire, to assess physicians’ knowledge and attitudes about OSA [11]. Several studies investigating knowledge and attitudes regarding OSA among physicians have been published to date. Among 92 cardiologists in the United States, 80% agreed that identifying patients at risk for OSA was very important, but only 18% felt confident in managing OSA patients [12]. Similarly, in a recent study, primary care physicians reported awareness of the importance of OSA, but only a few felt confident in managing OSA patients [13]. Also, 321 anesthesiologists in China felt they lacked adequate knowledge about OSA and had low confidence in managing OSA patients [14].

In Korea, the number of sleep studies has increased sharply since national health insurance coverage began in 2018. Korean patients with OSA are approached and treated differently by specialists in internal medicine, otolaryngology, neurology, and psychiatry. However, the participation rate of Korean pulmonologists in treating OSA patients is low compared to participation by other clinical departments. In this study, we investigated the knowledge and attitudes about OSA among pulmonologists in Korea.

This article was previously presented as a meeting abstract at the SLEEP 2024 meeting on June 3, 2024.

## Materials and methods

Study design

An online survey was performed in February 2023. A total of 634 respiratory specialists registered in the online directory of the Korean Academy of Tuberculosis and Respiratory Diseases were invited to participate. The survey used the OSAKA questionnaire, which was previously validated, employing its original English version without modification. We obtained permission to use the OSAKA questionnaire by contacting the Washington University School of Medicine and paying the required licensing fee. The survey was distributed along with a concise study overview, explicitly stating its anonymous nature and inviting participation. E-mails were sent to all participants requesting participation in the survey on three separate occasions. The study was approved by the Institutional Review Board (IRB) at Catholic Medical Center (UC24QISI0002).

Questionnaire

The OSAKA questionnaire consists of questions about knowledge and attitudes on OSA [11]. Its knowledge section is composed of 18 true-false statements, including five domains covering the epidemiology, pathophysiology, symptoms, diagnosis, and treatment of OSA. “Don't know” was included as a third response to minimize the effect of speculation. Separately, the attitude section of the OSAKA questionnaire contains five questions scored using a five-point Likert score; the first two questions are used to evaluate the importance of OSA, while the remaining three questions are used to assess confidence in its diagnosis and treatment. In this study, we additionally explored other variables, such as sex, age, degree, year of medical school graduation, year of specialization attainment, hospital distribution and classification, inpatient bed count, presence of a sleep center in the affiliated hospital, and OSA treatment experience.

Statistical analysis

The mean and standard deviation were computed for normally distributed continuous variables, and medi­an and interquartile range (25th-75th percen­tiles) were determined for non-normally distributed continuous data. Categorical data are presented as numbers and percentages. To compare clinical data be­tween two subgroups, Stu­dent’s t-test was performed for normally distributed data, while the Mann-Whitney U test was used for non-normally distributed data. To compare clinical data among groups, normally distributed data were subjected to a one-way analysis of variance with the Tukey post-hoc test. The Kruskal-Wallis test and Dunn post-hoc test were employed to compare non-normally distributed data. Categorical variables were compared using the chi-square or Fisher’s exact test, as appropri­ate. Pearson's correlation analysis was used to assess the relationship between knowledge and attitude scores. Statistical analyses were performed using R software (ver. 4.0.4; R Foundation for Statistical Computing, Vienna, Austria). P < 0.05 was considered significant in all analyses.

## Results

Baseline characteristics of the respondents

Of 634 pulmonologists queried, 127 (20%) completed the questionnaires, 87 of whom were men (68.5%) and 40 were women (31.5%) (Table 1). The mean age of respondents was 45.4 ± 8.6 years. According to academic background, 74 respondents (58.3%) had a PhD, which was higher than the 42 respondents (33.1%) with a Master’s degree or the 11 respondents (8.7%) with a Bachelor’s degree. Regarding the year of specialty acquisition, most pulmonologists obtained their specialty in the 1990s (n = 35, 27.6%) or 2000s (n = 50, 39.4%). The region of employment was mostly the Seoul capital area (n = 74, 58.3%), followed by Gyeongsang-do (n = 21, 16.5%) and Jeolla-do (n = 14, 11.0%). Considering medical care institutions, 91 (71.7%) respondents work at university hospitals, 27 (21.3%) work at general hospitals, and nine (7.1%) work at individual clinics. Approximately 80% of total respondents work at hospitals with >500 inpatient beds. Among the total respondents, 96 pulmonologists (75.6%) work at a hospital with a sleep center. However, only 71 (55.9%) respondents have experience managing patients with OSA.

**Table 1:** Baseline characteristics of the respondents Values are mean ± standard deviation or number of respondents OSA, obstructive sleep apnea

Characteristics	N = 127
Gender	
Male	87 (68.5%)
Female	40 (31.5%)
Age	45.4 ± 8.6
Degree	
Bachelor’s degree	11 (8.7%)
Master’s degree	42 (33.1%)
Doctor's degree	74 (58.3%)
The year of graduation from medical school	
1980~9	12 (9.4%)
1990~9	32 (25.2%)
2000~9	46 (36.2%)
2010~9	37 (29.1%)
The year of acquisition of the specialty	
1980~9	28 (22.0%)
1990~9	35 (27.6%)
2000~9	50 (39.4%)
2010~9	14 (11.0%)
Distribution of hospitals	
Seoul capital area	74 (58.3%)
Gangwon-do	4 (3.1%)
Chungcheong-do	10 (7.9%)
Gyeongsang-do	21 (16.5%)
Jeolla-do	14 (11.0%)
Jeju-do	4 (3.1%)
Classification of hospitals	
University hospital	91 (71.7%)
General hospital	27 (21.3%)
Individual clinic	9 (7.1%)
Number of inpatient beds	
No admission bed	6 (4.7%)
1 ≤ beds < 100	3 (2.4%)
100 ≤ beds < 300	9 (7.1%)
300 ≤ beds < 500	8 (6.3%)
500 ≤ beds	101 (79.5%)
Is there a sleep center in the working hospital?	
No	28 (22.0%)
Yes	96 (75.6%)
Don’t know	3 (2.4%)
Have you ever experienced patients with OSA?	
No	56 (44.1%)
Yes	71 (55.9%)

Knowledge

**Figure 1:**
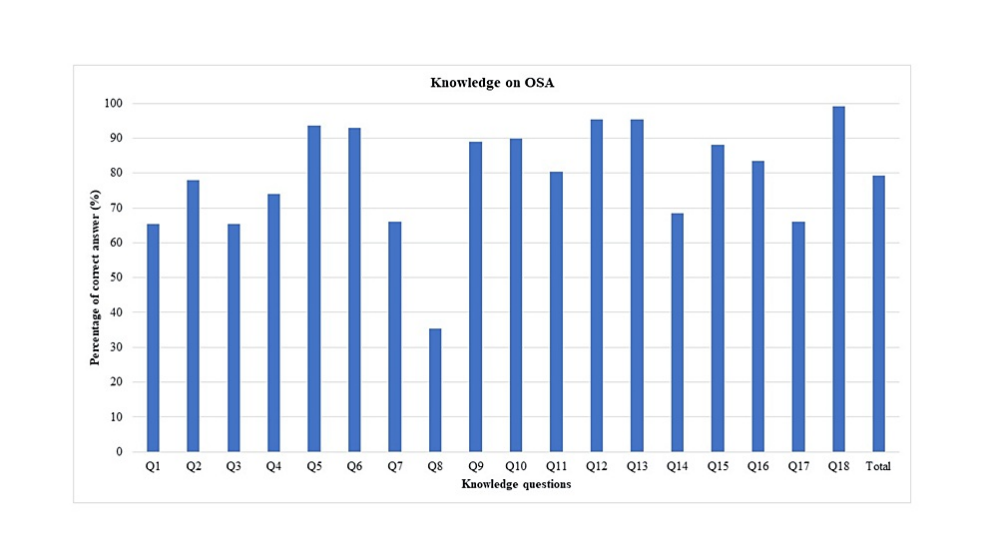
Percentage of correct answers to knowledge questions OSA, obstructive sleep apnea

Among 18 total questions, the average correct answers ratio was 80% (Figure 1). The highest percentage of correct answers (100%) was observed for question 18, which stated that there exists an association between cardiac arrhythmias and untreated OSA. The lowest percentage of correct answers (35%) was noted for question 8, which asked whether laser-assisted uvuloplasty is an appropriate treatment for severe OSA.

The mean (standard deviation (SD)) total OSA knowledge score was 15.0 (2.0). Table 2 shows the associations between socio-demographic factors and mean OSA knowledge score. Sex, year of specialty acquisition, and experience with OSA patients were significantly associated with the mean OSA knowledge score. Male respondents had significantly higher mean OSA knowledge scores compared to female respondents (15.0 vs. 14.0 points, P = 0.045). Those who acquired their medical specialty in the 1980s and 1990s showed higher mean OSA knowledge scores than those who acquired their specialty in the 2000s and 2010s. However, after post-hoc analysis, there was no significant difference between the four groups according to the decade of specialty acquisition. Respondents with experience treating OSA patients had significantly higher mean OSA knowledge scores than those without experience treating OSA patients (15.0 vs. 14.0 points, P = 0.034).

**Table 2:** Association between socio-demographic factors and means of knowledge score Values are medians and the IQR; p-values that are in italics are statistically significant (p < 0.05) IQR, interquartile range; OSA, obstructive sleep apnea

	N = 127	P-value
Gender		
Male	15.0 (14.0;16.0)	0.045
Female	14.0 (11.0;15.0)	
Age		
30-39	14.0 (13.0;15.0)	0.183
40-49	14.0 (13.0;16.0)	
50-59	15.0 (14.0;16.0)	
60-	15.0 (14.0;16.5)	
Degree		
Bachelor’s degree	15.0 (12.5;15.5)	0.345
Master’s degree	14.0 (13.0;16.0)	
Doctor's degree	15.0 (14.0;16.0)	
The year of graduation from medical school		
1980~9	15.0 (13.5;15.5)	0.066
1990~9	15.0 (14.0;16.0)	
2000~9	15.0 (13.0;16.0)	
2010~9	14.0 (12.0;15.0)	
The year of acquisition of specialty		
1980~9	15.0 (14.0;16.0)	0.043
1990~9	15.0 (14.0;16.5)	
2000~9	14.0 (13.0;16.0)	
2010~9	14.0 (12.0;15.0)	
Distribution of hospitals		
Seoul capital area	15.0 (13.0;16.0)	0.447
Gangwon-do	16.0 (13.0;16.5)	
Chungcheong-do	14.0 (13.0;15.0)	
Gyeongsang-do	14.0 (11.0;15.0)	
Jeolla-do	14.0 (13.0;16.0)	
Jeju-do	15.0 (14.5;15.5)	
Classification of hospitals		
University hospital	15.0 (13.0;16.0)	0.926
General hospital	15.0 (13.0;16.0)	
Individual clinic	15.0 (14.0;15.0)	
Number of inpatient beds		
No admission bed	14.5 (14.0;15.0)	0.634
1 ≤ beds < 100	15.0 (14.5;15.5)	
100 ≤ beds < 300	15.0 (14.0;17.0)	
300 ≤ beds < 500	13.5 (13.0;15.0)	
500 ≤ beds	15.0 (13.0;16.0)	
Is there a sleep center in the working hospital?		
No	14.0 (13.5;15.0)	0.125
Yes	15.0 (13.0;16.0)	
Don’t know	8.0 (7.0;11.5)	
Have you ever experienced patients with OSA?		
No	14.0 (12.0;15.0)	0.034
Yes	15.0 (14.0;16.0)	
Total score of 18-item questionnaire	15.0 (13.0;16.0)	

Attitude

Table 3 shows the associations between socio-demographic factors and mean attitude score. Age, year of graduation from medical school, year of specialty acquisition, and experience with OSA patients were significantly associated with mean OSA attitude score. Older respondents tended to have higher mean OSA attitude scores (P = 0.001); after post-hoc analysis, the mean OSA attitude score of respondents aged ≥60 years (19.8 ± 2.9 points) was significantly higher than that of respondents in their 30s (15.2 ± 3.6 points, P = 0.006) or 40s (17.0 ± 2.9 points, P = 0.040). Similar to this trend, groups with earlier graduation years tended to have higher mean OSA attitude scores (P = 0.011), although the difference was not significant in the post-hoc analysis.

**Table 3:** Association between socio-demographic factors and attitude score of OSA Values are mean ± standard deviation; p-values that are in italics are statistically significant (p < 0.05) OSA, obstructive sleep apnea

	N = 127	P-value
Gender		
Male	17.2 ± 3.2	0.138
Female	16.3 ± 3.3	
Age		
30-39	15.2 ± 3.6	0.001
40-49	17.0 ± 2.9	
50-59	17.7 ± 3.0	
60-	19.8 ± 2.9	
Degree		
Bachelor’s degree	16.5 ± 3.0	0.442
Master’s degree	16.5 ± 2.9	
Doctor's degree	17.2 ± 3.5	
The year of graduation from medical school		
1980~9	18.6 ± 3.1	0.011
1990~9	17.9 ± 3.0	
2000~9	16.7 ± 3.4	
2010~9	15.8 ± 3.1	
The year of acquisition of specialty		
1980~9	17.9 ± 3.0	0.005
1990~9	18.0 ± 2.8	
2000~9	15.8 ± 3.5	
2010~9	16.1 ± 3.2	
Distribution of hospitals		
Seoul capital area	17.1 ± 3.3	0.523
Gangwon-do	19.0 ± 1.8	
Chungcheong-do	16.7 ± 4.3	
Gyeongsang-do	16.1 ± 3.0	
Jeolla-do	17.4 ± 3.3	
Jeju-do	15.2 ± 2.5	
Classification of hospitals		
University hospital	16.8 ± 3.4	0.889
General hospital	17.2 ± 3.3	
Individual clinic	16.8 ± 1.2	
Number of inpatient beds		
No admission bed	16.2 ± 1.0	0.872
1 ≤ beds < 100	18.0 ± 0.0	
100 ≤ beds < 300	17.7 ± 3.3	
300 ≤ beds < 500	17.2 ± 4.1	
500 ≤ beds	16.8 ± 3.4	
Is there a sleep center in the working hospital?		
No	16.8 ± 3.3	0.358
Yes	17.0 ± 3.3	
Don’t know	14.3 ± 3.2	
Have you ever experienced patients with OSA?		
No	15.8 ± 3.5	<0.001
Yes	17.8 ± 2.8	
Total attitude score	16.9 ± 3.3	

For the year of medical specialty acquisition, the mean OSA attitude scores were significantly lower among those who acquired their specialty in the 2000s (15.8 ± 3.5) compared to those who acquired it in the 1980s (17.9 ± 3.0 points, P = 0.047) or 1990s (18.0 ± 2.8 points, P = 0.023). Overall, OSA attitude scores tended to be higher among older respondents and those who graduated from medical school and completed pulmonology specialist training earlier in their careers in Korea.

Table 4 shows the association between experience with OSA treatment and attitude. The total attitude score of the respondents with OSA treatment experience was 17.8 ± 2.8 points, significantly higher than that of respondents without OSA treatment experience (15.8 ± 3.5 points, P < 0.001). Attitudes toward the importance of OSA as a clinical disorder and identifying patients with OSA showed no significant differences according to OSA treatment experience. However, respondents with OSA treatment experience showed higher values. In particular, respondents with OSA treatment experience were significantly more confident in identifying at-risk patients, managing patients with OSA, and managing patients on continuous positive airway pressure than those without OSA treatment experience (P = 0.006, P < 0.001, and P = 0.003, respectively).

**Table 4:** Attitude score of OSA according to the experience of OSA treatment Values are mean ± standard deviation, number of patients or median (first quartile, third quartile). p-values that are in italics are statistically significant (p <0.05). OSA, obstructive sleep apnea; CPAP, continuous positive airway pressure

	Experience of OSA treatment			
	Yes	No	Total	P-value
	(N = 71)	(N = 56)	(N = 127)	
Importance of OSA as a clinical disorder	4.0 (3.0; 4.0]	3.0 (3.0; 4.0)	4.0 (3.0; 4.0)	0.419
Important to identify patients with OSA	4.0 (3.0; 4.0)	3.0 (3.0; 4.0)	3.0 (3.0; 4.0)	0.101
Confident identifying at-risk patients	4.0 (3.0; 4.0)	3.5 (3.0; 4.0)	4.0 (3.0; 4.0)	0.006
Confident managing patients with OSA	3.0 (3.0; 4.0)	2.0 (2.0; 3.0)	3.0 (2.0; 4.0)	<0.001
Confident managing patients on CPAP	4.0 (3.0; 4.0)	3.0 (2.0; 4.0)	4.0 (3.0; 4.0)	0.003
Total attitude score	17.8 ± 2.8	15.8 ± 3.5	16.9 ± 3.3	<0.001

Association between knowledge and attitude

When attitudes toward OSA were analyzed by knowledge scores, a significant correlation was seen (r = 0.38, P < 0.001) (Table 5). In addition, the knowledge score significantly correlated with responses to the five items of the attitude questionnaire.

**Table 5:** Correlations among attitude items and between attitudes and knowledge Pearson's correlation analysis was used to assess the relationship between knowledge scores and attitude scores. *p < 0.05 **p < 0.001 OSA, obstructive sleep apnea; CPAP, continuous positive airway pressure

	1	2	3	4	5	6
Importance of OSA as a clinical disorder	1					
Important to identify patients with OSA	0.83**	1				
Confident identifying at-risk patients	0.27*	0.32**	1			
Confident managing patients with OSA	0.09	0.18*	0.63**	1		
Confident managing patients on CPAP	0.18*	0.25*	0.43**	0.69**	1	
Knowledge score of 18-questionnaire	0.23*	0.24*	0.30*	0.30**	0.29*	1

## Discussion

In this study, we assessed knowledge and attitudes about OSA among pulmonologists in Korea. While previous studies have deployed the OSAKA questionnaire among various types of healthcare professionals, this was the first such study conducted among pulmonologists in Korea [12-16]. Globally, we were unable to find any research papers that have used the OSAKA questionnaire to specifically target pulmonologists. The main finding of our investigation is that the knowledge levels of Korean pulmonologists regarding OSA are comparable to or better than those reported in previous studies. Notably, pulmonologists who had experience managing OSA patients exhibited significantly higher knowledge and attitude scores than their counterparts without such experience. Interestingly, attitude scores tended to be higher among older respondents and those who had completed pulmonology specialist training earlier in their careers.

OSA impacts 3.2%-4.5% of the population in Korea and is linked to significant health complications and increased mortality rates [17]. Despite having sufficient access to healthcare services, up to 80% of patients with moderate or severe OSA remain undiagnosed [18,19]. Since the inclusion of polysomnography in the national health insurance coverage in 2018, there has been a sharp rise in the number of polysomnography examinations conducted in Korea. However, the field of OSA has traditionally been led by neurologists, otolaryngologists, and psychiatrists in Korea, with pulmonologists often playing a less prominent role. Based on a survey of the knowledge and attitudes of pulmonologists regarding OSA, our goal was to understand the current status of Korean pulmonologists in the management of OSA, aiming to increase interest and participation in sleep medicine among pulmonologists.

The relationship between OSA and cardiovascular diseases has been investigated in several studies [4,5,20]. However, OSA also has a significant impact on lung health. The effects of OSA on pulmonary diseases, such as COPD, lung cancer, and idiopathic pulmonary fibrosis, have been reported in several studies [9,21-24]. The co-existence of OSA and COPD, known as “overlap syndrome,” led to greater morbidity and mortality rates than either COPD or OSA alone [25]. An association between OSA and lung cancer has also been suggested by human and animal studies [26-28]. Intermittent hypoxia, swings in intrathoracic pressure, and recurrent collapse of the upper airway alter the anatomy and physiology of the respiratory system, leading to localized inflammation, structural changes, and increased reactivity [29-31]. Therefore, pulmonologists, as individuals who possess a thorough understanding of the respiratory system structure and physiology, actively treat respiratory illnesses, and are familiar with the use of equipment such as oxygen delivery systems and ventilators, offer numerous advantages and strengths in the treatment of OSA.

From our study, the total knowledge score calculated from the original 18 items of the OSAKA questionnaire was 15.0 (13.0-16.0) points, with an 80% correct answer ratio. Previous studies have reported score variations depending on the country, profession, and clinical department. Among cardiologists in the United States, the correct answer ratio was 76% [12]. Separately, a 60% correct answer ratio was reported among anesthesiologists in China and primary care physicians in Latin America [14,15]. Hence, although direct comparisons of knowledge scores between this study and others are challenging, the knowledge scores among the pulmonologists participating in this research were not substantially low. This may be due to the high proportion of pulmonologists working at university hospitals and increased concerns about sleep-breathing disorders. In Canadian otolaryngology, head and neck surgery residents, an exceptionally high knowledge score of 88.9% was recorded [32].

The correct response rate for question 8 in the knowledge section (laser-assisted uvuloplasty is an appropriate treatment for severe OSA: false) was notably low at 35%, in contrast to rates for other items. This pattern has also been observed in other studies, with one study demonstrating a correct answer rate of 33% [13]. However, Washington University, from which we obtained permission to use the OSAKA questionnaire, has indicated that this question should no longer be included in the most up-to-date version of the questionnaire. This suggests that the low correct answer rate observed in our study may not be significantly meaningful.

In the present study, respondents with experience treating OSA patients had higher OSA knowledge scores than those without experience treating OSA patients. This result is in agreement with those of several previous studies [13,33]. Physicians who have access to sleep centers had higher knowledge scores [33]. Physicians without experience in a department that manages OSA patients had lower OSA knowledge scores compared to those with such experience [13].

Our study indicated that higher OSA attitude scores were associated with older age, more years of practice, and prior experience with OSA patients. These results are similar to those of previous studies [12,13,15,34]. These findings imply that effectively managing OSA patients requires experience in clinical practice. The respondents without experience in OSA treatment had lower attitude scores regarding confidence in identifying at-risk patients and managing patients with OSA or those receiving continuous positive airway pressure therapy. This result reinforces the need for more education and training about OSA.

In the present study, we demonstrated a significant correlation between knowledge and attitude scores of OSA. Also, the knowledge score was associated with the five-item attitude questionnaire score. In a study of knowledge and attitudes of primary care physicians toward OSA in the Middle East and North Africa region, a positive but weak correlation between knowledge and attitude scores was noted [13]. Other studies have similarly reported correlations between attitude and knowledge scores [12,14].

As limitations of our study, first, despite sending three rounds of participation invitation emails to all respiratory specialists registered in the online directory of the Korean Academy of Tuberculosis and Respiratory Diseases within Korea, the study participation rate was not high at just 20%. Second, there is a possibility that the responses were biased toward pulmonologists who are familiar with or have a keen interest in OSA or sleep medicine. Third, the majority of pulmonologists in Korea work in university hospitals and general hospitals, resulting in >80% of respondents in this study being employed in hospitals with >500 beds, making it difficult to capture the opinions of pulmonologists working in smaller hospitals or private clinics. Finally, in Korea, some pulmonologists have access to sleep centers in their hospitals, and some do not. This aspect was not covered in our current study. However, considering that our results showed significantly higher knowledge and attitude scores among those with OSA management experience, it can be inferred that pulmonologists with access to sleep centers may exhibit higher knowledge and attitude scores.

## Conclusions

Consequently, this study represents the first investigation into the knowledge and attitudes about OSA among pulmonologists in Korea. The knowledge levels of Korean pulmonologists regarding OSA were comparable to or better than those reported in previous studies. Nevertheless, there remains a need for targeted education and practical exposure to OSA management, especially for younger respiratory physicians, to enhance their proficiency in treating OSA patients.
